# Assessing the oral toxicity of acetamiprid, spinosad, cypermethrin, and pyrethrins in the invasive hornet *Vespa velutina nigrithorax*

**DOI:** 10.1038/s41598-025-31988-x

**Published:** 2025-12-22

**Authors:** Paula Malaquias Souto, Soraia Sousa Santos, Artur Sarmento, Aline de Liz Ronsani, Ana Luísa Tomaz, Henrique M. V. S. Azevedo-Pereira, Sara Leston, Fernando Ramos, José Paulo Sousa, Nuno Capela

**Affiliations:** 1 Centre for Functional Ecology, Department of Life Sciences, Associated Laboratory TERRA, University of Coimbra, 3004-504 Coimbra, Portugal; 2 Department of Soils and Natural Resources, Universidade do Estado de Santa Catarina (UDESC Lages), 88520-000 Lages, SC Brazil; 3 Município de Cantanhede, Praça Marquês de Marialva, 3060-133 Cantanhede, Portugal; 4 Associated Laboratory for Green Chemistry (LAQV), Network of Chemistry and Technology (REQUIMTE), Apartado 55142, 4051-401 Porto, Portugal; 5 Faculty of Pharmacy, University of Coimbra, Polo III, Azinhaga de Santa Comba, 3000-548 Coimbra, Portugal

**Keywords:** Biological invasions, Chemical control, Ecotoxicology, Hymenoptera, Insecticide, Vespidae, Ecology, Ecology, Environmental sciences, Zoology

## Abstract

The yellow-legged hornet, *Vespa velutina* subs. *nigrithorax* Buysson, 1905, is an invasive species in Europe, posing substantial ecological and economic threats. Its biology and behavior, marked by rapid reproduction and aggressive predation, endanger native insects. This has negative impacts on agriculture, biodiversity, and human safety. Effective control is therefore essential to prevent and minimize its spread and ecological impact. This study assessed the acute oral toxicity of four commercial insecticide formulations containing acetamiprid, spinosad, cypermethrin, and a mixture of natural pyrethrins as active ingredients on *V. v. nigrithorax* workers. Our findings indicate that acetamiprid and spinosad are the most promising compounds, with acetamiprid inducing rapid mortality at low doses, and spinosad causing both lethal and sublethal effects, potentially disrupting hornet behavior and colony viability. Given that oral exposure generally resulted in higher toxicity than contact exposure, pest control strategies should incorporate multiple exposure pathways. Further investigations are necessary to confirm efficacy under field conditions and to evaluate potential ecological risks to non-target species. Overall, this study provides critical data to improve *V. v. nigrithorax* control and supports the development of more effective and environmentally sustainable control strategies against this invasive species.

**Supplementary Information:**

The online version contains supplementary material available at 10.1038/s41598-025-31988-x.

## Introduction

*Vespa velutina* subs. *nigrithorax* Buysson, 1905, originally native to Southeast Asia, was accidentally introduced into Europe in 2004 and reached northwestern Portugal by 2011^1,2^. Outside its native range, this species poses multiple threats: (i) ecologically, as a predator of honey bees and other pollinators, thereby affecting pollination services; (ii) economically, through negative effects on beekeeping and agriculture; and (iii) in public health, as a venomous insect frequently in close contact with humans that can cause severe allergic reactions and even fatalities^3^.

The successful expansion of *V. v. nigrithorax* can be attributed to its high adaptability and reproductive efficiency^3,4^. As a eusocial insect, this hornet forms large colonies divided into castes (queen/gynes, female workers, and males)^5^. After winter, the queen establishes a small primary nest, which expands as the population increases throughout the summer, leading to the construction of a larger secondary nest^6^. By the end of the summer, each nest can produce up to 300 gynes, which males can fertilize before hibernating during wintertime and starting the cycle again next year^6,7^.

Due to its rapid spread and significant ecological impacts, various physical, chemical, and biological control strategies have been investigated and proposed as potential control measures for this invasive species^8^. To capture adult hornets, baited traps, whether homemade or commercial, often use sugar- and protein-based substances that, after fermentation, lure hornets. However, these baits have low species specificity, thereby posing risks to other pollinators, and their overall impact on hornet colonies is limited^9^.

For nest elimination, the use of toxic baits, including protein- and sugar-based baits laced with insecticides, has gained increasing attention due to their potential to target foraging individuals and, indirectly, the entire colony^10,11^. These baits, either used to attract hornets to traps or placed directly into nests, are typically ingested by worker hornets and subsequently shared with larvae and other nestmates via trophallaxis, ultimately leading to colony collapse^10,12^. Nevertheless, toxic baits may once again attract other beneficial arthropods, such as insect pollinators. Alternatively, nests can be eliminated physically (e.g., by using fire) or through contact chemical treatments, in which insecticides are injected into the nest using extension poles. These methods are labor-intensive, require trained personnel, and are often unfeasible when nests are located high in trees or on buildings [6, 14, 45-47]. 

In both cases (i.e., use of toxic baits or insecticide injection), the hornet nests must still be removed to prevent contamination of non-target organisms, as pesticide residues can persist in the environment^13^.

Therefore, efforts are needed to mitigate and prevent the potential damage caused by *V. v. nigrithorax* control strategies, prioritizing environmentally sustainable and species-specific strategies. The absence of species-specific control protocols and the lack of formal approval for targeted biocidal products at the European level have resulted in the adoption of ad hoc mitigation strategies, often extrapolated from approaches developed for other pest taxa^14–16^. For example, in Portugal, the insecticides currently recommended are non-selective, broad-spectrum formulations, intended for general wasp control^17^, highlighting the lack of targeted management strategies for this invasive species^14^. To date, the only study conducted at the European level has been that of^14^, which assessed the acute contact toxicity of four commercial pesticide formulations under laboratory conditions. However, no studies have yet addressed the oral toxicity of pesticides in the invasive hornet, reinforcing the need for further investigation.

In this context, assessing the toxicity of commonly used pesticide formulations is crucial, as it can improve the efficiency of nest elimination by enabling more accurate dosage regulation and reducing environmental impacts^18,19^. Hornets may ingest pesticides not only during nest treatments but also through baited traps containing toxic substances; therefore, evaluating oral exposure is essential. Although the use of toxic baits remains controversial due to low selectivity and potential off-target effects, understanding oral exposure and feeding acceptance remains ecologically relevant. Therefore, we performed acute oral toxicity tests on adult *Vespa velutina nigrithorax* under controlled laboratory conditions, using four commercially available formulations of insecticides containing acetamiprid, spinosad, cypermethrin, and a mixture of natural pyrethrins as active ingredients. To our knowledge, this is the first formal report on acute oral toxicity in this species, providing a foundation for future comparisons between exposure routes.

## Material and methods

### Pesticide selection and characterization

Four insecticidal formulations were selected based on the work developed by^14^: Starpride Max [AC; active ingredient (a.i.) acetamiprid], Spintor (SP; a.i. spinosad), Cythrin 10EC (CY; a.i. Cypermethrin), and Pirecris (PI; mixture of natural pyrethrins as a.i.) (Table 1). For clarity and readability, pesticides are hereafter referred to by the name of their active ingredient or its abbreviation.

**Table 1:** Insecticides tested in the study. insecticide general information, physico-chemical characteristics, and relevant ecotoxicity data^20,21^.

Commercial formulation	Starpride max	Spintor	Cythrin 10EC	Pirecris
Active ingredient (a.i.)	Acetamiprid	Spinosad	Cypermethrin	Pyrethrin mix (cinerin I & II, jasmolin I & II, and pyrethrin I & II)
Concentration	200 g a.i./L; 17.6% (w/w)	480 g a.i./L; 2.14% (w/w)	100 g a.i./L; 10.9% (w/w)	20 g a.i./L; 44% (w/w)
Acute oral LD_50_ (honey bee)	14.53 μg a.i. bee⁻^1^	0.057 μg a.i. bee⁻^1^	0.172 μg a.i. μg bee⁻^1^	0.95 μg a.i. bee⁻^1^
Acute oral LD_50_ (mammals)	146 mg a.i./kg	> 2000 mg a.i./kg	287 mg a.i./kg	700 mg a.i./kg
Short-term dietary LD50 (birds)	> 741 mg kg body weight⁻^1^ day⁻^1^	> 5156 mg kg feed⁻^1^	> 5620 mg kg feed⁻^1^	-
DT_50_ (lab at 20 °C) (soil)	1.6 days	15 days	117.7 days	2.5 days

### Collection and exposure of hornets

Acute oral toxicity assays on adult *V. v. nigrithorax* workers were performed following protocols adapted from the standardized OECD guidelines for honey bee and bumblebee acute oral toxicity tests^22,23^. Adult workers were collected from nine active secondary nests located in different districts of Portugal during September and October 2024, which corresponds to the typical secondary nest season (late summer to winter; S1 Table). For each tested pesticide, hornets from 5 different nests were used, totaling 50 individuals tested per pesticide dose (10 hornets per nest × 5 nests). Each nest was treated as an independent source of individuals, and hornets from different colonies were never intermixed across treatments.

Adult hornets were captured at the nest entrance with a sweep net as they emerged and placed in 1.5 L plastic containers for transport. In the laboratory, the containers were chilled at −20 °C until the hornets were immobilized for safe handling. The chilling duration varied depending on the time required to reduce activity. Subsequently, individuals were sexed and weighed, and females weighing 300–500 mg were selected, as they most likely correspond to worker castes, the main component of the colony^6^.

Each hornet was individually caged in 500 cm^3^ transparent plastic cages perforated with approximately 2 mm holes to ensure adequate ventilation. Hornets were placed in the cages one day prior to testing and maintained under controlled conditions for acclimation (25 ± 2 °C; 60% ± 20% RH; constant darkness; forced air circulation). Non-contaminated food was provided ad libitum through 35 mm Petri dishes containing a mixture of water, honey, and agar–agar (1:0.25:0.0065) to prevent starvation. Prior to oral pesticide exposure, hornets were subjected to a fasting period of 2–3 h. After fasting, each hornet received 20 µL of either the pesticide solution or sugar solution (control) using a micropipette, ensuring the complete consumption of the provided volume. Instances of avoidance behavior, when observed, were recorded and are reported in the Results section. After exposure, non-contaminated food was returned to the hornets, and cages were placed back in the incubator. Mortality and abnormal behavior were recorded 4–6 h post-exposure and subsequently every 24 h (24, 48, 72, and 96 h, when applicable). Each experiment was terminated by freezing the test cages (with individuals inside) at ≤ −20 °C.

After exposure, each individual was observed for approximately 30 s, and behavior was classified into categories adapted from standard ecotoxicological guidelines and preliminary observations^14^. The behavioral categories included: affected (impaired coordination or body contractions), apathy (reduced responsiveness and prolonged immobility), cramps (one or more legs appearing paralyzed), hyperactivity (increased activity relative to controls), locomotion difficulties (impaired coordination or inability to walk properly, often stumbling or dragging legs), and moribund (loss of mobility with weak leg or antennal movements, typically preceding death).

### Preparation of test concentrations and pesticide residue analysis

Eight doses were defined for each pesticide based on previously available data for *Vespa velutina nigrithorax*^14^, complemented by acute toxicity information for other Hymenoptera species available in the Pesticide Properties DataBase (PPDB) and Bio-Pesticides DataBase (BPDB)^20,21^. Preliminary range-finding tests were then conducted to refine the concentration series for each product, ensuring a mortality gradient from 0 to 100% within the 96-h exposure period (Table 2). Ten hornets were tested per treatment, including one control and eight pesticide concentrations per test. Stock and test solutions for each treatment were prepared using a 50% (w/w) sugar solution. Pesticide concentrations were verified using standard analytical methods: gas chromatography–tandem mass spectrometry (GC–MS/MS) for the mixture of natural pyrethrins samples, high-performance liquid chromatography with ultraviolet detection (HPLC–UV) for acetamiprid and spinosad samples, and ultra performance liquid chromatography–mass spectrometry (UPLC–MS) for cypermethrin.

**Table 2:** Pesticide concentrations (mg a.i./mL) in test solutions used for oral exposure of yellow-legged hornet *Vespa velutina nigrithorax* adults.

AC	SP	CY	PI
0.0011	0.0058	0.1850	0.0371
0.0016	0.0094	0.2590	0.0742
0.0022	0.0145	0.3626	0.1484
0.0030	0.0240	0.5076	0.2969
0.0042	0.0383	0.7106	0.5938
0.0059	0.0613	0.9949	1.1875
0.0082	0.0981	1.3929	2.3750
0.0114	0.1570	1.9500	4.7500

The reported values are the outcomes of the pesticide residue quantification analytical techniques. AC: acetamiprid); SP: spinosad; CY: cypermethrin; PI: mixture of natural pyrethrins.

### Statistical analysis

To estimate the lethal concentrations and doses required to eliminate 50% (LC/LD_50_) and 90% (LC/LD_90_) of the tested population (expressed in mg a.i./mL and µg a.i./hornet), data were analyzed using a generalized linear model (GLM) with a probit link function, following the OECD guidelines for acute toxicity testing^22^. Each pesticide was analyzed independently within its respective concentration range. The models were fitted in R^24^ using the *glm()* function from the ‘stats’ package, and lethal dose and concentration estimates (LD₅₀ and LD₉₀) with 95% confidence intervals were obtained using the *dose.p()* function from the ‘MASS’ package. Model goodness of fit was assessed based on deviance and pseudo-R^2^ values. Pairwise comparisons between LC₅₀ and LC₉₀ values at 24 h were performed using the Wald test via the delta method, based on asymptotic normal approximations of probit GLM estimates. All analyses were performed in R using the ‘tidyverse’ and ‘stats’ packages. Mortality data, along with their means and standard errors, were calculated using R, and the ‘ggplot2’ package^25^ was used to generate the graphs presented in the Results section.

## Results

### Mortality and lethal doses

**Fig. 1:**
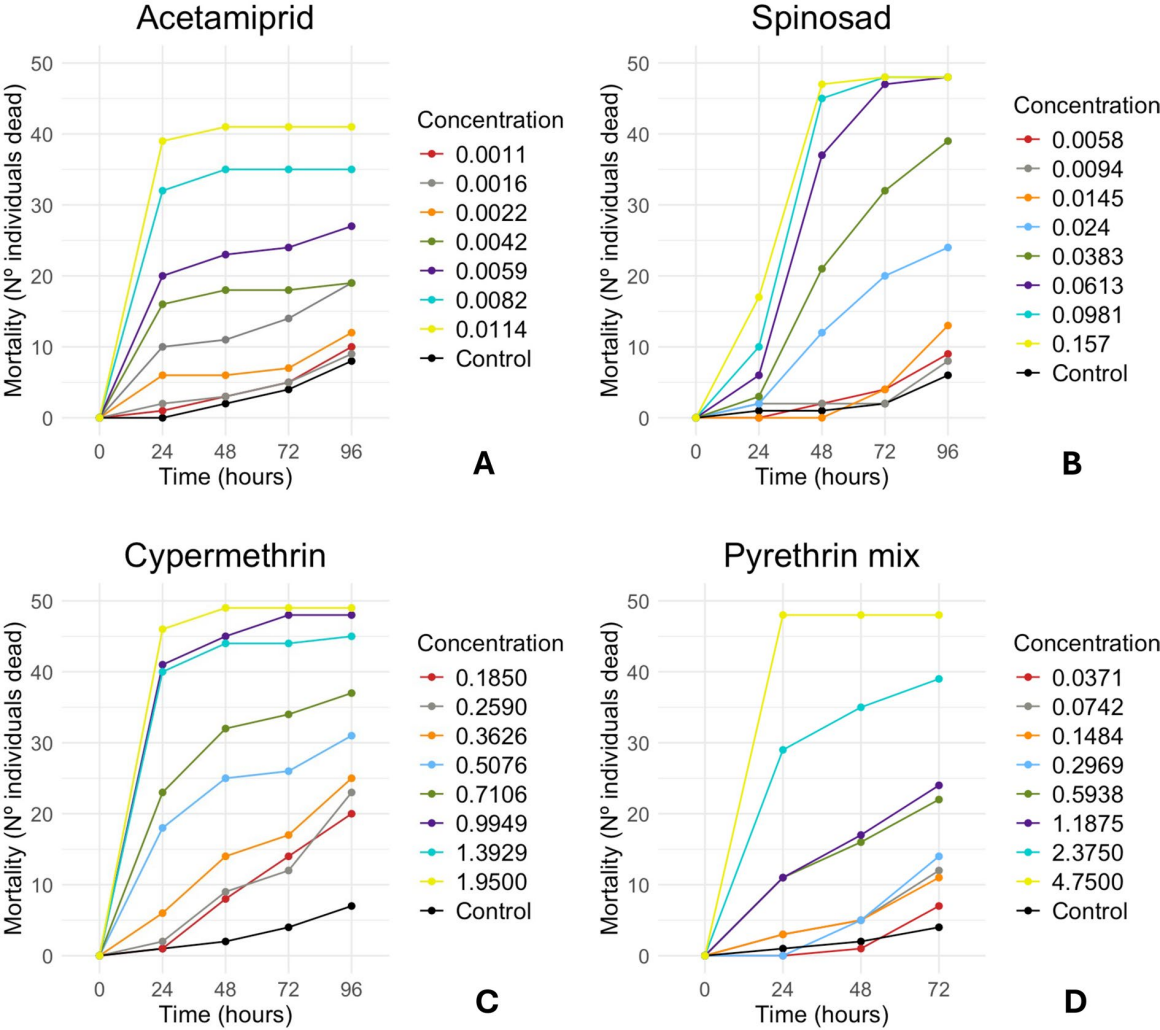
Mortality (number of individuals dead) of *Vespa velutina nigrithorax* orally exposed to 20 μL of four different pesticides. **A**–**D**, mortality curves for acetamiprid (**A**), spinosad (**B**), cypermethrin (**C**), and a mixture of natural pyrethrins (**D**). concentrations are in mg a.i/mL.

Lethal concentration and dose estimates (LC/LD₅₀ and LC/LD₉₀, 95% CI) at 24 h are presented in Table 3. Complete GLM probit results for all exposure times (24–96 h), including confidence intervals and model fit statistics, are provided in Supplementary Table S6. At later time points (48–96 h), for most products, the probit models yielded unrealistically high or undefined LC/LD estimates due to complete or near-complete mortality across all tested doses. In such cases, reliable curve fitting is not feasible because intermediate responses (e.g., around 50% mortality) were absent. Consequently, only the 24 h data representing the most consistent and biologically meaningful estimates were retained for the main comparisons. The acute oral toxicity of the four compounds—acetamiprid (AC), spinosad (SP), cyermethrin (CY), and mixture of natural pyrethrins (PI)—to *Vespa velutina nigrithorax* varied markedly among compounds (Table 3 and Fig. 1).

**Table 3:** Oral lethal toxicity (LC/LD_50_ and LC/LD_90_) after 24 h of oral formulation exposure to the yellow-legged hornet *Vespa velutina nigrithorax.*

Insecticide	LC50	LC90	LD50	LD90
	(mg a.i./mL)	(mg a.i./mL)	(μg a.i./hornet)	(μg a.i./hornet)
**AC**				
24 h	0.0064 (0.0056, 0.0073)	0.0170 (0.01275, 0.0131)	0.1277 (0.1123, 0.1450)	0.3380 (0.2615, 0.4360)
**SP**				
24 h	0.4098 (0.1952, 0.8572)	3.5544 (0.8381, 15.0446)	8.1908 (3.9013, 17.1268)	70.8014 (16.7026, 299.5118)
**CY**				
24 h	0.7195 (0.6481, 0.7978)	1.6862 (1.4205, 2.0219)	2.2399 (1.4270, 3.4876)	109.2322 (30.1959, 394.5226)
**PI**				
24 h	1.6695 (1.3201, 2.1099)	9.1219 (5.7624, 14.4339)	33.3885 (26.4026, 42.1972)	182.4132 (115.2360, 288.6258)

Values are presented in lethal concentration (LC) and lethal dose (LD), in mg a.i./mL and μg a.i./hornet, respectively. the values in parentheses indicate the 95% confidence interval values for LD and LC. a.i.: active ingredient. AC: acetamiprid; SP: spinosad; CY: cypermethrin; PI: mixture of natural pyrethrins.

Acetamiprid consistently exhibited the highest toxicity throughout the entire observation period (AC, Table 3; Fig. 1A). LC/LD₅₀ and LC/LD₉₀ values remained low, indicating a rapid and potent toxic effect shortly after exposure. According to the mortality curves, 0.0114 mg a.i./mL resulted in the highest mortality, affecting 41 out of 50 individuals after 48 h of exposure.

Spinosad demonstrated increasing toxicity over time, with LC/LD values decreasing steadily from 24 to 48 h (SP, Table 3 and S6; Fig. 1B). This suggests a time-dependent effect of the active ingredient, with mortality increasing over time even at lower concentrations. Mortality reached its maximum after 72 h of exposure at concentrations of 0.0981 and 0.157 mg a.i./mL (48/50 individuals). The same level of mortality was also achieved at 0.0059 mg a.i./mL after 96 h of exposure.

Cypermethrin showed intermediate toxicity (CY, Table 3; Fig. 1C). This formulation caused the highest number of deaths (49 out of 50 individuals). However, a strong repellent effect was observed, particularly at concentrations ≥ 0.5076 mg a.i./mL—the fourth highest concentration tested. During exposure, repellent behavior was evident in most individuals at or above this level. To assess toxicity, feeding had to be administered forcibly. The highest mortality was observed at 1.9500 mg a.i./mL, causing mortality from the early stages up to 96 h.

The mixture of natural pyrethrins was the least toxic among the tested pesticides, with relatively high LC/LD values compared to the other formulations (PI, Table 3), indicating lower toxicity. However, high mortality was still recorded, affecting 48 out of 50 individuals, the same maximum value as observed for spinosad, within the first 24 h (Fig. 1D). This level of mortality was only achieved at the highest tested concentration (4.7500 mg a.i./mL). The mixture of natural pyrethrins did not fulfill the required criteria to continue testing up to 96 h, as control mortality exceeded 20% at that time. Consequently, lethal dose estimates were only calculated for 24, 48, and 72 h.

Pairwise comparisons of LC₅₀ and LC₉₀ values (24 h) indicated significant differences in oral toxicity among the four tested pesticides (Supplementary Table S7). Acetamiprid was the most potent compound, requiring significantly lower doses to achieve both 50% and 90% mortality compared with the other formulations (p < 0.001). Spinosad and cypermethrin showed comparable toxicity (p > 0.1), whereas both were considerably more toxic than the natural pyrethrins formulation (p < 0.001). Overall, the toxicity ranking was acetamiprid > spinosad ≈ cypermethrin > natural pyrethrins, consistent across LC₅₀ and LC₉₀ endpoints.

All insecticides caused concentration-dependent mortality. However, differences in toxicity profiles were evident: acetamiprid acted rapidly but caused the lowest overall mortality among the tested compounds (82%); spinosad exhibited delayed yet increasing toxicity, reaching 96% mortality; cypermethrin showed a moderate and consistent effect, achieving the highest mortality rate (98%) but also exhibited a strong repellent effect; and the mixture of natural pyrethrins displayed the weakest toxic response, with high mortality (96%) observed only at the highest concentrations. In all cases, the highest tested concentrations consistently led to the greatest mortality, reinforcing the concentration-dependent nature of the toxic effects.

### Abnormal behaviors

After the fasting period, during pesticide exposure, all hornets readily accepted the test solutions and showed no abnormal behaviors, indicating good palatability of the tested formulations. The only exception was cypermethrin, which elicited avoidance behavior, confirming its previously reported repellent properties.

The most prevalent behavioral categories were “affected” and “moribund” hornets. Overall, “locomotion difficulties” were observed for all pesticides. Nonetheless, only hornets exposed to cypermethrin presented detached or broken legs, particularly the tibia and tarsus. Occasionally, “hyperactivity” was observed in hornets exposed to spinosad and in one individual to the mix of natural pyrethrins. “Apathy” was mainly observed in individuals exposed to spinosad, while only one individual showed this behavior when exposed to acetamiprid.

## Discussion

Under field conditions, when attempting to eliminate nests through pesticide injection, yellow-legged hornets may be exposed to the product via contact and/or oral routes. While contact toxicity has been characterized for some products^14^, there is still a lack of data on the effects of oral exposure. In the current study, we evaluated the acute oral toxicity of four commercial insecticide formulations on *Vespa velutina nigrithorax*, aiming to simulate a broader exposure scenario by providing data on an alternative route of pesticide action. To allow comparisons and to complement existing data, we used the same formulations previously tested for acute contact toxicity. Our findings revealed clear differences in toxicity profiles and suggest that the formulations with acetamiprid and spinosad as active ingredients are the most effective, in line with previous findings by contact exposure. This suggests that these compounds may be a promising solution to effectively eliminate hornet nests.

Acetamiprid showed a rapid toxic effect, with significant mortality occurring within the first 24 h post-exposure. It exhibited consistently low LC/LD values for the four time points, indicating a fast mechanism of toxicity. However, LC_90_ estimates exceeded the highest tested concentration, suggesting that higher concentrations may be necessary to fully characterize the dose required to achieve 90% mortality at all time points. Mortality peaked within 24 h post-exposure at relatively low concentrations, consistent with acetamiprid’s role as an agonist of nicotinic acetylcholine receptors, which disrupts neural transmission after ingestion^26^. This neonicotinoid insecticide is generally considered safer than other compounds of the same class, showing comparatively low toxicity to pollinators such as bumblebees and honey bees^27^. For instance, acute oral exposure of *Bombus terrestris* to acetamiprid resulted in an LD_50_ of 300 μg a.i./bee over 24 h^28^, approximately 1,000 times higher than the LD_50_ obtained in the present work (0.1277 μg a.i./hornet). Similarly^27^, reported 48 and 72 h LD_50_ values of 13.13 and 12.88 μg a.i./bumblebee, respectively, about 100 times greater than the LD_50_ obtained in the present work. These differences suggest that hornets may lack the detoxification capacity observed in bumblebees, which can better metabolize cyano-based neonicotinoids, such as acetamiprid^29^.

Spinosad exhibited a distinct toxicity profile. Unlike acetamiprid, this microbial-derived insecticide caused progressively increasing mortality over time, with significant effects observed at low concentrations. Notably, it also presented the highest incidence of sublethal effects, which could be deleterious in a natural context, affecting adult hornets’ activities and, consequently, nest health^30^. This pattern of delayed toxicity and pronounced sublethal effects is consistent with its known mode of action, as an allosteric modulator of nicotinic acetylcholine receptors and acting on gamma-aminobutyric acid (GABA) receptors [30, 33, 38, 48-50]. .

Despite being a natural pesticide, known to present low toxicity for mammals^31^, it presents high toxicity for pollinators^30,32,33^. The oral toxicity of spinosad has already been tested on honey bees and bumblebees, presenting an LC_50_−24 h of 0.00734 mg a.i./mL and an LC_50_−72 h of 0.0080 mg a.i./mL, respectively^30,32^. Therefore, spinosad is much more toxic to these pollinators than to the yellow-legged hornet, which presented an LC_50_ of 0.4098 mg a.i./mL at 24 h.

Cypermethrin induced the highest mortality among all tested formulations. However, a notable repellent effect was observed at concentrations above 0.5076 mg a.i./mL, compromising the likelihood of ingestion under natural conditions. Although this study does not advocate the use of toxic baits—given their low species-specificity and associated ecological risks—understanding acceptance or avoidance behaviors remains essential for accurately interpreting oral exposure results and assessing non-target risks in scenarios where hornets may encounter contaminated food sources. Accordingly, the strong repellency of cypermethrin suggests that oral intake under field conditions would likely be limited, meaning that the observed laboratory LC values may overestimate its real-world efficacy. In such a context, data from contact exposure assays should be prioritized when evaluating the practical application potential of this compound^14^. Similar repellent activity has been reported in other insect species. For instance, cypermethrin-based solutions have been used as positive controls in repellency tests involving houseflies (*Musca domestica* L.), and a 10% cypermethrin-ethyl alcohol solution demonstrated strong avoidance effects against the American cockroach (*Periplaneta americana* L.)^34,35^. Conversely, in pollinators such as honey bees, this active ingredient has shown high toxicity but no significant repellency effects^36,37^.

Although exposure of the yellow-legged hornet to the mixture of natural pyrethrins led to high mortality and sublethal effects, such as locomotion difficulties and hyperactivity, the applied concentrations were substantially higher than those of the other tested pesticides, leading to significantly higher LD and LC values. These lethal and sublethal effects are consistent with pyrethrins’ mode of action, which targets the insect nervous system by binding to the sodium channels of heart, nerve, and skeletal muscle cell membranes, prolonging their opening, leading to hyperexcitation and ultimately death^38^. However, considering the product’s formulation (20 g a.i./L) and its highest recommended application rate (225 mL of product per hL of water), if a hornet ingested 20 µL of this spray solution, it would receive approximately 0.9 µg of active ingredient—about 35 times lower than the 24 h LD₅₀ estimated in our assays (33 µg per individual). Like cypermethrin, pyrethrins act on the same receptor and are more toxic to honey bees than yellow-legged hornets^39^.

A comparative analysis between the yellow-legged hornet and the honey bee further emphasizes differences in species susceptibility. The acute oral LD₅₀ values reported for honey bees are 14.53, 0.057, 0.172, and 0.95 µg a.i./bee for acetamiprid, spinosad, cypermethrin, and natural pyrethrins (mix), respectively. In contrast, the corresponding LD₅₀ values obtained in this study for *V. v. nigrithorax* were 0.1277, 8.1908, 2.2399, and 33.3885 µg a.i./hornet. The resulting honey bee–hornet LD₅₀ ratios indicate that *V. v. nigrithorax* is approximately 114 times more sensitive to acetamiprid than honey bees, whereas the latter are 144-, 13-, and 35-fold more sensitive to spinosad, cypermethrin, and pyrethrins, respectively. These fold differences reveal contrasting sensitivity patterns among formulations, indicating that acetamiprid exhibits greater selectivity toward the target species, while spinosad, cypermethrin, and pyrethrins pose greater relative risk to pollinators. This interspecific comparison reinforces the need to balance efficacy against *V. v. nigrithorax* populations with potential ecological side effects when considering field applications.

Our data show that the tested compounds differ in both efficacy and onset of action, with important implications for environmental impact. Among the evaluated formulations, acetamiprid and spinosad emerged as the most promising active ingredients for effective pest management and control at low doses. These findings reinforce the conclusions drawn in our previous work. By contrast, although cypermethrin and pyrethrins also caused high mortality, their repellent effects and the high concentrations required, respectively, raise practical concerns in a real-world context.

Within the European Union, pesticide use is strictly regulated due to its environmental impact and negative effects on non-target species^40^. According to the European Food Safety Agency (EFSA), the recommended agriculture application rate for acetamiprid is 80–100 g a.i./ha as a good agricultural practice^41^. Although our study focuses on acute oral toxicity under controlled laboratory conditions rather than field application scenarios, a theoretical comparison with EFSA reference rates helps contextualize the magnitude of the doses tested.

In a hypothetical scenario, considering the LD_90_−24 h value for acetamiprid obtained in this study (0.3380 μg a.i./hornet) and assuming an average nest with 2,000 adult hornets^6^, approximately 0.00068 g of acetamiprid would be sufficient to eliminate the nest, assuming all individuals ingested the formulation. Based on reported nest densities in Coimbra, Portugal (1–3 nests per 100 ha between 2020 and 2025^42^), the total amount of acetamiprid hypothetically required per 100 ha would range from 0.00068 to 0.0020 g. This is several orders of magnitude lower than the standard agricultural rate of 8,000–10,000 g for the same area^41^.

In mainland Portugal, approximately 140,000 nests were identified and eliminated between the species’ arrival and 2023. Following the same theoretical rationale, this would correspond to roughly 102 g of acetamiprid, equivalent to the quantity used to spray about one hectare in agriculture. Thus, nest control using acetamiprid could be achieved with only a small fraction of the quantities applied in standard agricultural practices across Europe. This comparison is not intended to suggest that acetamiprid should be applied as a spray, but rather to illustrate the relatively small amount that would be required if hornets were exposed through oral ingestion under controlled or field-relevant conditions. Such information is important to better understand potential exposure routes and to estimate realistic environmental risks associated with pesticide use. Nevertheless, additional compounds effective against this hornet should be evaluated to enable the rotation of active ingredients and prevent potential resistance development.

In this study, oral exposure proved to be more toxic than contact, with significantly lower LC and LD values for all tested formulations, except for the mixture of natural pyrethrins^14^. These findings are consistent with previous studies on other hymenopteran species, such as honey bees, where oral toxicity has also been shown to exceed contact toxicity (e.g^43^). This difference may indicate variations in adsorption, distribution, metabolism, and/or excretion between contact and oral exposure for the active ingredient tested^43^ and should be considered when assessing their overall effectiveness. Although the general toxicity patterns observed for both routes were similar, oral exposure data provide essential complementary information by revealing how the compounds behave when ingested rather than absorbed through the cuticle. In addition to these mechanistic differences, oral toxicity results are also relevant from a management perspective. Since both contact-based (nest injection) and oral-based (toxic baits) approaches are currently used in the control of *Vespa velutina nigrithorax*, comparing their toxicological profiles is crucial to determine which approach offers the best balance between efficacy, selectivity, and environmental safety. These findings highlight the importance of integrating both exposure routes when evaluating pesticide performance together to better estimate field efficacy. In other words, under real-world conditions, where both contact and oral exposures occur simultaneously (as in nest injection), lower pesticide concentrations may be sufficient to eliminate nests compared with values predicted from contact- or oral-only studies. Then, in cases where hornets are only exposed orally (toxic baits), both acetamiprid and spinosad appear to be promising alternatives. Nevertheless, it is imperative to test these compounds in real-world scenarios, to ensure pesticide stability and verify that baits are indeed visited only by this invasive species.

Although this study does not aim to promote the use of insecticide-treated baits, our findings contribute to improving current control practices by providing scientifically grounded data on the oral toxicity of compounds already employed in nest treatments. In many regions, local authorities commonly inject insecticides directly into nests and subsequently leave them in situ. By identifying compounds that are effective at lower doses and less toxic to non-target organisms, our results support the refinement of these existing practices, helping to reduce the amount of pesticide use while maintaining efficacy. This evidence-based approach may thus guide more sustainable management strategies for *Vespa velutina nigrithorax*, ensuring effective nest elimination with minimized environmental impact. Furthermore, pesticide management strategies should be adjusted to prevent the development of resistance, since insects have evolved multiple mechanisms against insecticides via both contact and oral routes^44^. To obtain more robust conclusions, further studies should combine both exposure routes under laboratory conditions, followed by field validation.

## Conclusions

This study provides critical insights into the acute oral toxicity of four commercial insecticide formulations targeting the invasive yellow-legged hornet, *Vespa velutina nigrithorax*, enhancing our understanding of exposure pathways involved in the chemical control of this invasive species. Our results demonstrate that formulations containing acetamiprid and spinosad are the most effective at inducing mortality, corroborating previous contact exposure findings and highlighting their potential as promising control agents. Overall, oral exposure was more toxic than contact exposure for most tested compounds, underscoring the importance of considering multiple exposure routes to accurately assess pesticide efficacy under field conditions. Nevertheless, certain limitations should be acknowledged, including the use of individual hornets rather than whole colonies, and testing commercial formulations instead of pure active ingredients. Future research should integrate both exposure routes, include field-based trials and larval bioassays, and evaluate potential effects on non-target organisms to ensure environmental safety. These findings provide a valuable basis for developing integrated, evidence-based management strategies for the control of this invasive species, which poses serious ecological, economic, and public health threats.

## Supplementary Information

Supplementary Information 1

Supplementary Information 2

Supplementary Information 3

Supplementary Information 4

Supplementary Information 5

Supplementary Information 6

Supplementary Information 7

## Data Availability

The datasets generated during and/or analyzed during the current study are all available as Supplementary Material.
